# Why Men Don’t Marry

**Published:** 1892-04

**Authors:** 


### WHY MEN DON'T MARRY.

The cultivated bachelor of thirty-three has not by any means lost his taste for the society of women; but beauty is no longer all powerful, and he is attracted by good sense and solid qualities as well. Men of his sort know what terrible bores ignorant girls can be, how utterly unreasonable they often are, and how much more liable they are in middle life to grow acrid, snappish, or positively ill-tempered. There is no one so perverse as the woman without intellectual interests, whose situation happens to be at variance with her ideas of comfort, or who, being comfortable, is conscious of the faint contempt, or rather slight avoidance, of those around her. Women are perfectly well aware when men listen from politeness alone, and those among them to whom that lot falls grow as bitter as some disappointed spinsters. Companionship is impossible between the able and the silly. Thoughful men, too, are aware that it is the clever girls, not the simpletons, who are free from the senseless extravagance which is, perhaps, of all foibles which are not exactly vices, the most permanently irritating in wives. That thing, at least, culture has done for the majority of cultured women—it has taught them how to count. The immense majority of cultivated girls are economical. Frugality is the road to independence. They could not live their lives if they cost their fathers too much, and they learn to know the value of money, and to avoid debt with horror. They are not, perhaps, devoted to "housekeeping," as some of the unlettered are, meaning, three times out of five, endless and harassing interference with their servants; but they can keep house, when they know their incomes, at an outlay well within them. Men know what it is to be bored. There is no bore on earth equal to the woman who can neither talk nor listen, who has no mental interest in common with her husband. It is true that cultured girls may be too frank of speech and contradict a man too openly, causing him to think that she is trying to "put him down." The habit is a mere gesture in reality, a colt's kick of pleasure in the free field, and not a sign of vicious temper ; but it constantly ruins a bright girl's chances, and has done much to create in society an impression which is, on the evidence of facts, entirely unfounded. Cultivated girls have, in fact, a trick of thinking that argument is conversation, and that contradiction shows mental fearlessness—a trick which men, even tolerant men, never quite like.

